# Response and adaptation of photosynthesis, respiration, and antioxidant systems to elevated CO_2_ with environmental stress in plants

**DOI:** 10.3389/fpls.2015.00701

**Published:** 2015-09-10

**Authors:** Zhenzhu Xu, Yanling Jiang, Guangsheng Zhou

**Affiliations:** ^1^State Key Laboratory of Vegetation and Environmental Change, Institute of Botany, Chinese Academy of SciencesBeijing, China; ^2^Chinese Academy of Meteorological SciencesBeijing, China

**Keywords:** abiotic stress, antioxidant system, elevated CO_2_, drought, global warming, photosynthesis, respiration

## Abstract

It is well known that plant photosynthesis and respiration are two fundamental and crucial physiological processes, while the critical role of the antioxidant system in response to abiotic factors is still a focus point for investigating physiological stress. Although one key metabolic process and its response to climatic change have already been reported and reviewed, an integrative review, including several biological processes at multiple scales, has not been well reported. The current review will present a synthesis focusing on the underlying mechanisms in the responses to elevated CO_2_ at multiple scales, including molecular, cellular, biochemical, physiological, and individual aspects, particularly, for these biological processes under elevated CO_2_ with other key abiotic stresses, such as heat, drought, and ozone pollution, as well as nitrogen limitation. The present comprehensive review may add timely and substantial information about the topic in recent studies, while it presents what has been well established in previous reviews. First, an outline of the critical biological processes, and an overview of their roles in environmental regulation, is presented. Second, the research advances with regard to the individual subtopics are reviewed, including the response and adaptation of the photosynthetic capacity, respiration, and antioxidant system to CO_2_ enrichment alone, and its combination with other climatic change factors. Finally, the potential applications for plant responses at various levels to climate change are discussed. The above issue is currently of crucial concern worldwide, and this review may help in a better understanding of how plants deal with elevated CO_2_ using other mainstream abiotic factors, including molecular, cellular, biochemical, physiological, and whole individual processes, and the better management of the ecological environment, climate change, and sustainable development.

## Introduction

The major components of climate change include elevated atmospheric carbon dioxide concentrations (elevated CO_2_), warming, and altered precipitation patterns, as well as their interactions within and with other environmental factors (IPCC, 2013). Based on updated information, with increases in global atmospheric CO_2_ concentrations of 43% from the pre-industrial level of 280 μmol mol^-1^ in 1750 to the present level of 400 μmol mol^-1^ (an annual increase of 1.35%), the global CO_2_ concentration has increased by about 1.55 ppm CO_2_ per year over the past 55 years. It continues to be elevated at an unprecedented pace of ∼1.0 μmol mol^-1^ per year, as a result of the further increase in the cumulative emissions of CO_2_ to the atmosphere during the 21st century (400 μmol mol^-1^ in 2011 vs. 936 μmol mol^-1^ in 2100; IPCC, 2013; NASA, 2014). Meanwhile, the global mean surface temperature is expected to increase by 2.6–4.8°C by the end of the 21st century (2081–2100), relative to the 1986–2005 level under RCP8.5, based on a more undisciplined management scenario with higher greenhouse gas emissions (IPCC, 2013). The climate changes, such as elevated CO_2_, rising temperature, and altered precipitation, have resulted in drastic impacts on the natural ecosystems, such as in vegetation function, sustainable food production, and crop yields (Lobell et al., 2011; Peñuelas et al., 2013; Ruiz-Vera et al., 2013; Xu et al., 2013a, 2014; Lavania et al., 2015), leading to more profound impacts when the climate changes are combined with other environmental constraints, such as air pollution, nutrition limitation, and their interactions (Gillespie et al., 2012; Peñuelas et al., 2012; Xu et al., 2013a,b; Wang et al., 2015).

Herein, we focus on the critical biological processes of plants with regard to climate change, including (mainly) photosynthesis, respiration, the antioxidant system, and the related metabolic activities. Photosynthesis and respiration are two fundamental physiological processes of plants, because the former involves initial carbon fixation, light energy transfer, and oxygen release, and the latter works on carbon eﬄux, energy production, and the relevant substrate metabolisms, such as those providing the carbon skeleton. They play a critical role in balancing the carbon budget and maintaining the carbon sink in terrestrial ecosystems, as well as in the response and feedback to climate change (Melillo et al., 1993; Prentice et al., 2001; Sage, 2004; Long et al., 2006; Atkin et al., 2010; Atkin, 2015). The excessive accumulation of reactive oxygen species (ROS) often occurs in plants grown under abiotic stress, while an enzymatic and non-enzymatic antioxidant defense system may work to protect plants against oxidative stress-induced damage, which can be affected by climate change (such as elevated CO_2_, drought, and heat waves; Pérez-López et al., 2009; Gill and Tuteja, 2010; Xu et al., 2014; Zinta et al., 2014; Way et al., 2015).

Rising CO_2_ has affected almost all crucial biological processes, including photosynthesis, respiration, and antioxidant systems, as well as other key secondary metabolisms in plants (Poorter et al., 1997; Long et al., 2004; Matros et al., 2006; Peñuelas et al., 2013; Singh and Agrawal, 2015). All other effects of elevated CO_2_ on individual plants and ecosystems may be partly derived from these fundamental biological responses (Long et al., 2004; Ainsworth and Rogers, 2007; Peñuelas et al., 2013; Zinta et al., 2014). Genetic variations relative to the biological processes’ traits might also be impacted by elevated CO_2_, closely linking to these responses in various spatiotemporal aspects, from molecular, biochemical, and physiological, through individual levels and ecosystems, up to the entire Earth’s life system, interacting with multiple environmental factors (both biotic and abiotic) as well as human-driven disturbances at different temporal scales (Long et al., 2004; Teng et al., 2009; Peñuelas et al., 2012, 2013; Jagadish et al., 2014; Zinta et al., 2014).

As stated above, plant responses to climate change have become a hot topic in botanical research across various scales in the recent decades. Many reports have reviewed the biological responses to CO_2_ enrichment, and their interactions with environmental change, including photosynthesis and stomatal behavior (e.g., Long et al., 2004; Ainsworth and Long, 2005; Ainsworth and Rogers, 2007). Our earlier review by Xu et al. (2013a) examined plant growth, carbon and nitrogen (N) allocations, gas exchange responses to elevated CO_2_ with drought and high temperature. Although this review discussed the changes in growth and photosynthesis, and water use efficiency (WUE) in higher plants exposed to CO_2_ enrichment with abiotic variables, the various underlying mechanisms of the critical biological processes that are affected, modulated, and controlled by elevated CO_2_ with other abiotic environmental variables were not fully covered, particular at the molecular, organelle, cell, biochemical, physiological, organ, individual, and ecosystem scales. Actually, no systematic synthesis of these has been well reviewed, thus far. Therefore, in this review, based on correcting and synthesizing any new progress of the relevant research concerning plant biology and climatic change, we attempted to systematically summarize the considerable study results that have reported the responses of photosynthesis, respiration, and the antioxidant systems, as well as the key substrate metabolisms to elevated CO_2_ with other environmental variables. Particularly, we reviewed the underlying mechanisms and the response pathways, as well as their interrelationships. Finally, the future perspectives for this study related to the possible implications are briefly presented and discussed. Thus, the present review may be of current interest in terms of its interdisciplinary and systematic synthesis, providing comprehensive information on the important historical and new experimental results, relative theoretical analysis, underlying mechanisms, and potential applications to promote further research.

## Responses of Critical Biological Processes to Elevated CO_2_

### Photosynthetic Response to Elevated CO_2_ Concentrations

#### Response Magnitude

The responses of photosynthesis to elevated CO_2_ concentrations have been reviewed in many reports [e.g., Drake et al., 1997 most for enclosure results; Long et al., 2004; Nowak et al., 2004; Ainsworth and Long, 2005; Ainsworth and Rogers, 2007 for free-air CO_2_ enrichment (FACE)]. The stimulation of the light-saturated photosynthetic CO_2_ assimilation rate (*A*_sat_) is a general response to CO_2_ enrichment, with an average of 31% in the FACE experiments (Ainsworth and Rogers, 2007), and 23–58% in the potted plant experiments from earlier reports (Ryle et al., 1992; Drake et al., 1997). The magnitude of the stimulation by CO_2_ enrichment varies with the different plant functional types (PFTs), with a maximum for trees and C_3_ grasses; moderate for shrubs, C_3_ and C_4_ crops, and legumes; and minimum for C_4_ grass (even with a negative response; Drake et al., 1997; Ainsworth and Long, 2005; Ainsworth and Rogers, 2007). Therefore, there is greater variation in the stimulation by elevated CO_2_, depending on the plant species, PFTs, and their surroundings, specifically environmental conditions like nutrition and water resource availability. For instance, elevated CO_2_ leads to an increase in the *A*_sat_ of *Arabidopsis thaliana* leaves by 82%, since the N availability is ample (Markelz et al., 2014). However, a recent study of soybean plants indicated that elevated CO_2_ did not produce significant effects on midday net photosynthetic rate (*A*_net_), either in the FACE or open-top chamber (OTC) studies (Bunce, 2014), suggesting that the *A*_net_ at high photosynthetic photon flux density (PPFD) might be limited by a low ribulose 1, 5-bisphosphate carboxylase/oxygenase (Rubisco) carboxylation capacity (Bunce, 2014). Actually, other abiotic and biotic factors such as high temperature (e.g., Ruiz-Vera et al., 2013), drought (Xu et al., 2014), N deficit (Markelz et al., 2014), genetic variation (Ainsworth et al., 2004), and leaf senescence (Liu et al., 2014) may also diminish the photosynthetic response to elevated CO_2_.

The same results appeared in *Lolium perenne* and *Medicago lupulina* plants in controlled chambers (Farfan-Vignolo and Asard, 2012). Moreover, C_4_ plants may have no response to elevated CO_2_, because their CO_2_ concentrating mechanism (CCM) may concentrate the CO_2_ 12–20 times at the site of Rubisco, which is relatively higher than in C_3_ species (von Caemmerer and Furbank, 2003; Ainsworth and Rogers, 2007). Case studies confirmed this theoretical conclusion under well-watered conditions in either enclosure (e.g., Xu et al., 2014) or FACE experiments (e.g., Leakey et al., 2006; Markelz et al., 2011). However, under a water deficit, the stimulation of the C_4_ *A*_sat_ by elevated CO_2_ still appears, because the drought-induced impairment of C_4_ photosynthesis might be ameliorated by elevated CO_2_ (Markelz et al., 2011; Meng et al., 2014; Xu et al., 2014). Moreover, C_4_ plants can avoid photorespiration to promote CO_2_ fixation with higher light use efficiency (von Caemmerer and Furbank, 2003; Long et al., 2006). On the other hand, the down-regulation of the photosynthesis capacity is also more profound in C_3_ species than in C_4_ species (Morgan et al., 2001; Duarte et al., 2014), due in part to the N dilution, possibly because C_3_ plants need to invest more N from the leaf into Rubisco, relative to the C_4_ species, so that the former may easily undergo more severe N dilution under CO_2_ enrichment (Sage et al., 1987; Yin, 2002; Luo et al., 2004; Sage, 2004), with no CCM (von Caemmerer and Furbank, 2003).

In addition to N limitation, photosynthetic acclimation under higher CO_2_ levels may result from high stomatal and internal resistances, higher starch levels, and diluted chlorophyll concentrations (Delucia et al., 1985; Teng et al., 2009). Under elevated CO_2_, carbohydrate accumulations, such as starch size and number of chloroplasts (Teng et al., 2006, 2009), can be enhanced, partially due to the carbon substrate increase. However, the excessive carbohydrate accumulation may cause feedback inhibition or physical damage at the chloroplast level, reducing the photosynthetic capacity (Delucia et al., 1985; Aranjuelo et al., 2011). More importantly, the Rubisco response, excessive sugar feedback, and the related gene expression may, together; play crucial roles in plants’ photosynthetic acclimation under higher CO_2_ concentrations, particularly for long-term CO_2_ enrichment under a nitrogen availability deficit (see details below).

#### Molecular Mechanisms: Role of Rubisco

The stimulation of photosynthesis in C_3_ species by short-term elevated CO_2_ has been well established, and confirmed under almost all experimental conditions, particularly with FACE (e.g., Long et al., 2004; Ainsworth and Rogers, 2007; Duarte et al., 2014). However, with long-term exposure to elevated CO_2_ or other limitations, photosynthetic acclimation or the down-regulation of the photosynthetic capacity may occur, depending on the species, plant developmental stage, and environmental conditions (Moore et al., 1999; Urban et al., 2012; Sanz-Sáez et al., 2013).

**FIGURE 1:**
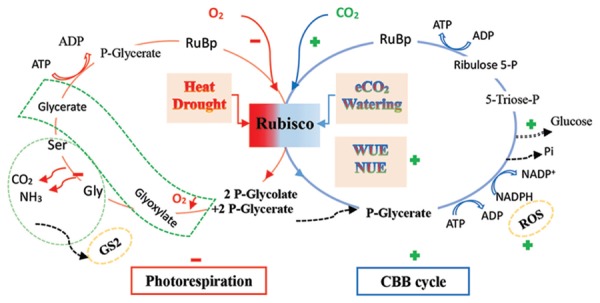
**A diagrammatic outline of the Calvin–Benson–Basshan (CBB) cycle and photorespiration pathway in plants in response to elevated CO_2_ with abiotic factors**. Rubisco has two sites of carboxylation and oxygenation. Elevated CO_2_ (eCO_2_) may promote carboxylation, but repress oxygenation under ample environmental conditions, such as well-watering, whereas extreme abiotic stress, such as heat and drought, may repress carboxylation but promote oxygenation (Wingler et al., 1999; Tingey et al., 2003). An energy consumption trade-off between the key cycles may occur, possibly modified by the CO_2_ level, in which photorespiration may be promoted to quench reactive oxygen species (ROS), related to glutamine synthetase (GS2) to recycle ammonia, diminishing photo-oxidation and photo-inhibition (dotted orange line ellipse; Kozaki and Takeba, 1996; Watanabe et al., 2014). A low Gly:Ser ratio provides evidence that photorespiration is repressed in eCO_2_ (Kebeish et al., 2007). Water use efficiency (WUE) and nitrogen use efficiency (NUE), despite the N dilution, should be enhanced by elevated CO_2_, by decreasing stomatal conductance and investing relatively more N into the Rubisco protein (Palmroth et al., 2013). The photorespiration process is compartmentalized into the chloroplast (red line ellipse), peroxisome (dotted green line bent rectangle), and mitochondrion (dotted green line ellipse). The green plus and red minus signs denote the stimulation and suppression via rising CO_2_, respectively (mainly referring to Kozaki and Takeba, 1996; Wingler et al., 1999; Tingey et al., 2003; Ainsworth and Rogers, 2007; Moroney et al., 2013; Xu et al., 2013a; Watanabe et al., 2014).

Rubisco has been identified as a controlling rate enzyme for carbon fixation (Eichelmann et al., 2009). Here, we succinctly summarize the five major mechanisms that might explain the response to elevated CO_2_, involving Rubisco: (1) under current CO_2_ concentration levels, although the value of the Rubisco Michaelis–Menten constant (*K*_m_) for CO_2_ is close to the current intercellular CO_2_ concentration (*C*_i_) (c. 190 μmol mol^-1^) at the site of carboxylation (von Caemmerer and Evans, 1991; Ainsworth and Rogers, 2007). CO_2_, as a substrate of photosynthesis, does not have to reach saturation; therefore, the rising CO_2_ can lead to an immediate increase in the Rubisco carboxylation velocity, due to an increase in the carbon substrate availability. (2) The Rubisco catalyzing function has two intrinsic side features: carboxylation and oxygenation. The carboxylation rate is ∼2.2 fold greater than the oxygenation rate at 25°C in C_3_ plants; that is, about one-third of the ribulose-1,5-bisphosphate (RuBP) may be consumed in the oxygenation reaction (Ainsworth and Rogers, 2007). Thus, elevated CO_2_, as a competing substrate, can competitively inhibit the oxygenation of RuBP (light-dependent photorespiration) through the down-regulation of Rubisco’s affinity for O_2_, while competitively promoting the carboxylation of RuBP via the up-regulation of Rubisco’s affinity for CO_2_ (Bowes, 1991; Long, 1991; Ainsworth and Rogers, 2007; Kane et al., 2013; Moroney et al., 2013). Consequently, this leads to the stimulation of photosynthesis, which may be compromised by heat and drought due to the enhancement of Rubisco’s affinity for O_2_ (Wingler et al., 1999; Tingey et al., 2003; Carmo-Silva et al., 2008; Moroney et al., 2013) (**Figure 1**). On the other hand, (3) with continually increasing CO_2_, the ATP products may not meet enough of the demand for RuBP regeneration, and a reduction in Rubisco’s activation state may occur, usually accompanied by a decrease in the capacity for RuBP regeneration, as well as in the RuBP pool, as indicated by a decline in the ATP:ADP ratio in the chloroplast (Eichelmann et al., 2009; Watanabe et al., 2014). (4) A reduction in the Rubisco content via N dilution, particularly under long-term elevated CO_2_, may finally contribute to the reduction of carboxylation at the Rubisco active site. In addition, the nitrogen use efficiency (NUE) might be increased due to the optimization of the resource use (Moore et al., 1999; Luo et al., 2004; Fukayama et al., 2012; Urban et al., 2012; Palmroth et al., 2013; Sanz-Sáez et al., 2013). Because the leaf N of C_3_ species can be more invested in Rubisco (more than 25% vs. 10–15% of the leaf N in C_3_ and C_4_ plants, respectively), the former may be affected more profoundly by N dilution.

Finally, (5) Hexokinase (HXK), as a sensor of excessive photosynthate, may be involved in the downregulation of the Rubisco content (Ainsworth and Rogers, 2007; Kirschbaum, 2011; see below). In summary, with respect to the Rubisco response, parts (1) and (2) above may explain the stimulation of photosynthesis by elevated CO_2_, while the last three points may provide a mechanism for understanding the downregulation of the photosynthetic capacity under relatively long-term elevated CO_2_ or other resource deficit conditions, such as a scarcity of N.

#### Sugar Feedback Inhibition of Photosynthesis

Under higher CO_2_ concentrations, a prevailing explanation of the downregulation of photosynthesis may be ascribed to the sugar feedback inhibition hypothesis: certain reactive bioprocess activities within the Calvin–Benson–Basshan (CBB) cycle may be inhibited by elevated CO_2_, due to the overload of the chemical reaction substrates. The hypothesis of the sugar feedback mechanism suggests that excessive photosynthate in chloroplasts under elevated CO_2_ may trigger the sugar signal network (HXK acting as a flux sensor) to down-regulate the Rubisco contents through the gene expression processes, affecting the subunit of Rubisco (Drake et al., 1997; Stitt and Krapp, 1999; Long et al., 2004; Ainsworth and Rogers, 2007).

As noted above, HXK acting as a flux sensor in mesophyll cells may involve the down-regulation of the Rubisco content associated with genetic expressions under elevated CO_2_; however, plants may prefer to reduce the Rubisco activity relative to the RuBP regeneration capacity (Ainsworth and Rogers, 2007). For instance, Aranjuelo et al. (2011) found a decline in wheat Rubisco and its activase protein content accompanying a photosynthetic down-regulation. In addition, the effects of the source-sink balance in response to CO_2_ enrichment may play an important role in the regulation of the photosynthetic capacity.

Based on the “source-sink” hypothesis, some plants with a strong sink can encounter photosynthetic down-regulation, to some extent, under higher CO_2_, which can generally be repressed by other limitations, such as intrinsic genetic constraints or the specific plant developmental stage (such as the flowering stage; Lewis et al., 2002; Ainsworth et al., 2004). Moreover, when enhanced carbohydrate availability exceeds the plants’ ability to fully utilize carbohydrates, due to nutrient or inherent internal growth limitations, the feedback may lead to a lower level of photosynthesis (Kirschbaum, 2011), which may lead to an imbalance in the carbon sink:source ratio (Bryant et al., 1998; Aranjuelo et al., 2011). For instance, wheat plants exposed to high atmospheric CO_2_ are incapable of excessive accumulation of leaf photoassimilate, due to the lack of an increase in the carbon sink strength (Aranjuelo et al., 2011).

Furthermore, the respiratory ATP may be consumed more under elevated CO_2_ (Watanabe et al., 2014); for example, the rate of the carbohydrate/sugar export (i.e., the cost related to the carbohydrate export) is higher under elevated CO_2_ than under normal CO_2_ (Watanabe et al., 2014), which may cause a negative feedback effect on photosynthesis. This also highlights the close link between the photosynthetic and respiratory bioprocesses, between both the CBB and tricarboxylic acid (TCA) cycle, under climate change (Moroney et al., 2013; Watanabe et al., 2014).

### Response of Respiration to Elevated CO_2_

#### Photorespiration

Photorespiration enables the photosynthetic process to recycle the phosphoglycolate produced by the oxygenase reaction of Rubisco, consequently avoiding more carbon loss, with some protective regulation functions for plants, such as in the oxidative defense mechanism (Bowes et al., 1971; Husic et al., 1987; Kozaki and Takeba, 1996; Wingler et al., 1999; Carmo-Silva et al., 2008; Moroney et al., 2013). However, as reported in the review by Ainsworth and Rogers (2007), at room temperature (25°C), photorespiration can lead to a loss of 23–30% of the carbon fixed by photosynthesis with the rising temperature, whereas the CO_2_ fixation may be increased by ∼53% if only the carboxylation reaction occurs, without the oxygenation reaction (Monteith, 1977; Long et al., 2006).

Widely accepted results show that photorespiration can be restricted when C_3_ plants are grown under high CO_2_ concentrations (Bowes, 1991; Tingey et al., 2003; Long et al., 2004), because in C_3_ plants, the carboxylation capacity of Rubisco, with a low catalytic activity (operating below its *K*_m_ for CO_2_), is easily promoted by high CO_2_. Meanwhile, an increase in the CO_2_ concentration, leading to a high CO_2_:O_2_ ratio, may reduce its oxygenation reaction capacity, inhibiting photorespiration (Bowes, 1991; Tingey et al., 2003; see above). For example, based on the earlier report by Sharkey (1988), the photorespiration rate should fall by ∼50% when the CO_2_ level is doubled. In *A. thaliana* plants grown under elevated CO_2_, although the accumulations of several major amino acids (including glutamate, aspartate, asparagine, and alanine) were enhanced, a lower level of glycine (Gly), an intermediate of photorespiration, was observed in the plants, leading to a decline in the Gly:Ser ratio, indicating a lower photorespiration activity (Kebeish et al., 2007; **Figure 1**).

Enhanced photoperoxidation in chloroplasts can induce a destruction of the chlorophyll and a disassembly of the chloroplast membranes, leading to a decline in photosynthesis (Heath and Packer, 1968). Conversely, the constraints of photorespiration by elevated CO_2_ may also reduce the H_2_O_2_ products, weakening oxidation stress, possibly protecting the photosynthetic apparatus (Watanabe et al., 2014; Zinta et al., 2014). Based on the fact that photorespiration has a protective function against photo-oxidation (Kozaki and Takeba, 1996; Zinta et al., 2014), possibly via the up-regulation of glutamine synthetase (GS2) to recycle ammonia, diminishing photo-oxidation and photo-inhibition (Kozaki and Takeba, 1996). This brings with it another dilemma: a decline in photorespiration under rising CO_2_ levels may cancel the protective role, leading to a higher level of photo-oxidation than the higher rate of carboxylation stimulated by elevated CO_2_ can maintain. In order to solve this dilemma, further research is required to cope with climate change, possibly by manipulating the modulated photorespiration bioprocess (Moroney et al., 2013).

#### Mitochondrial Respiration

Mitochondrial respiration involves the carbon balance in the whole plant, with 20–80% of the carbon fixed in photosynthesis being released again through the respiration process. The respiration of the leaves in both the light and dark can account for ∼50% of the whole-plant respiratory CO_2_ (Ayub et al., 2014). The response of dark leaf respiration (*R*_d_) to elevated CO_2_ remains debatable, with a decrease in the major reports, while increasing or remaining stable in a number of experiments (e.g., Ryle et al., 1992; Curtis and Wang, 1998; Drake et al., 1999; Amthor, 2000; Gonzalez-Meler et al., 2004; Loreto et al., 2007; Ayub et al., 2014). For instance, there is a 15–18% range in the reduction of foliar respiration when plants are grown under a doubled CO_2_ concentration, relative to the ambient CO_2_ level from one review (Drake et al., 1999; references). However, no significant response to the leaf *R*_d_ was observed in *L. perenne* plants exposed to high CO_2_; although, the leaves grown in elevated CO_2_ had a relative lower *R*_d_ (Ryle et al., 1992). A small response in the leaf respiration rate to a short-term CO_2_ elevation (a 1.5% decrease) was obtained from the deciduous tree species used in an earlier experiment by Amthor (2000), with similar evidence found in soybean plants from a recent report by Ayub et al. (2014). Thus, for the plants grown under elevated CO_2_, the *R*_d_ decrease response is *general*, not *universal*.

Correspondingly, the underlying mechanism has also been proposed in two contrasting hypotheses: elevated CO_2_ may enhance the *R*_d_ due to the great increase in the respiratory substrates, such as sugar; whereas the N dilution induced by elevated CO_2_ might reduce the demand on dark respiration to support the protein turnover, leading to a decline in the *R*_d_ (Thomas et al., 1993; Gonzalez-Meler et al., 2004; Fukayama et al., 2011; Markelz et al., 2014). A recent report showed that CO_2_ enrichment can accelerate the accumulation of the relevant carbohydrates, such as sugar, starch, and respiratory glycolysis intermediates like hexose-P, phosphoglycerate (PGA), and phosphoenolpyruvate (PEP) in *A. thaliana* plants, which may enhance the respiration potential (Watanabe et al., 2014). Recent evidence has also indicated that the promotion to greater photo-assimilation availability at elevated CO_2_ leads to a great transcriptional up-regulation of the genes, in association with the respiratory pathway (Leakey et al., 2009a; Fukayama et al., 2011; Markelz et al., 2014), supporting the first hypothesis. However, this may depend on the availability of the nutritional components, including nitrogen in the plants and/or the soil. For instance, based on a recent report by Markelz et al. (2014), widely and greatly adaptive responses of the expression of the respiratory genes were obtained when the plants were exposed to elevated CO_2_. However, the transcriptional reprogramming with the stimulation of leaf respiration by elevated CO_2_ can be suppressed by limited nitrogen availability (Markelz et al., 2014).

### Response of Antioxidant System to Elevated CO_2_

**FIGURE 2:**
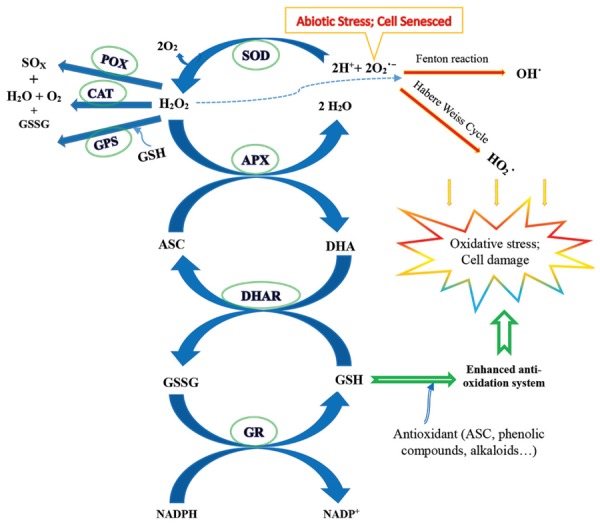
**A diagrammatic outline of the antioxidant defense systems and the responses to elevated CO_2_ with abiotic stress**. Elevated CO_2_ may alleviate the damage of oxidative stress from abiotic stress factors, such as heat, drought, and ozone, by ameliorating the antioxidant defense systems of non-enzymatic compounds, potentially including ascorbate (ASC), glutathione (GSH), phenolic compounds, and alkaloids, and the relevant enzymes, possibly including superoxide dismutase (SOD), ascorbate peroxidase (APX), dehydroascorbate reductase (DHAR), glutathione reductase (GR), peroxidase (POX), catalase (CAT), and glutathione peroxidase (GPX). ROSs, including superoxide radicals ($O_{2}^{\cdot -}$), hydrogen peroxide (H_2_O_2_), hydroxyl radicals (OH^⋅^), and perhydroxy radicals ($H\,O_{2}^{\cdot }$), accumulate when plants undergo abiotic stress or are senesced by the Fenton reaction and/or the Habere Weiss mechanism (Hodges and Forney, 2000; Gill and Tuteja, 2010). Whether the rising CO_2_ mitigates oxidative damage and the response magnitude, and which parts play major roles, depends on the plant species, crop varieties, developmental stage, abiotic factors, and their combinations (e.g., Hodges and Forney, 2000; Gill and Tuteja, 2010; Abd Elgawad and Asard, 2013; Kumari et al., 2013; Zinta et al., 2014). GSSG, oxidized glutathione; DHA, dehydroascorbate. This diagram is based mainly on the studies by Gill and Tuteja (2010) and Zinta et al. (2014).

The ROS in plants, including superoxide radicals ($O_{2}^{\cdot -}$), hydrogen peroxide (H_2_O_2_), the hydroxyl radical (OH^⋅^), and the perhydroxy radical ($H\,O_{2}^{\cdot }$), often accumulate when plants are subjected to abiotic stress, while the antioxidant defense system with enzymatic and non-enzymatic machinery may work to protect the plants against damage due to oxidative stress. This occurs particularly in the face of stressful environmental changes, such as adverse climatic changes like droughts and heat waves (**Figure 2**) (Schwanz and Polle, 1998; Pérez-López et al., 2009; Gill and Tuteja, 2010; Sekmen et al., 2014; Zinta et al., 2014). Generally, when plants become senesced, with some antioxidants increasing and others decreasing, the ROS may accumulate in a large amount, and the antioxidant system does not work well. This is often indicated by enhanced lipid peroxidation and decreased levels of antioxidant enzymes, such as superoxide dismutase (SOD) and catalase (CAT), leading to programmed cell death (PCD), particularly under severe abiotic stress (Dhindsa et al., 1981; Hodges and Forney, 2000; Gill and Tuteja, 2010; Duarte et al., 2013).

Elevated CO_2_ may increase the levels of antioxidants, including polyphenols, ascorbate (ASC), alkaloids, and some antioxidant enzyme activities (such as CAT and SOD), with a significant enhancement in the antioxidant capacity, leading to declines in the ROS levels (Mishra and Agrawal, 2014; Zinta et al., 2014). For example, when the plants were exposed to elevated CO_2_, increases in the ASC and phenol levels were obtained in *Beta vulgaris* (Kumari et al., 2013), and increases in the ASC, glutathione (GSH), and ASC/GSH, as well as in their redox status, were found in *L. perenne* and *M. lupulina* (Farfan-Vignolo and Asard, 2012). Ascorbate synthesis can be triggered and enhanced by excessive carbohydrate production due to elevated CO_2_ (Smirnoff and Wheeler, 2000; Zinta et al., 2014), which is closely linked to carbon metabolism (Smirnoff and Wheeler, 2000), and together improve the plant-antioxidant defense system. Moreover, with a delay in the onset of senescence and/or severe stress under elevated CO_2_ conditions, it is suggested that the antioxidant profiles, such as the accumulation of antioxidant compounds and antioxidant enzyme activity, may show better performance in dealing with the biological process of senescence (Hodges and Forney, 2000). For instance, a reduction in the oxidative stress under elevated CO_2_ was found in *Zingiber officinale* (Ghasemzadeh et al., 2010), *Catharanthus roseus* (Singh and Agrawal, 2015), a temperate grassland shrub, *Caragana microphylla* (Xu et al., 2014), a bean, *Vigna radiate* (Mishra and Agrawal, 2014), and *A. thaliana* plants (Zinta et al., 2014).

However, with the elevated CO_2_-alleviated oxidation stress evidence coming from a number of reports (e.g., Pérez-López et al., 2009; Xu et al., 2014), these results have not been confirmed in some species, such as in *Spinacia oleracea* leaves (Hodges and Forney, 2000). Farfan-Vignolo and Asard (2012) reported that CO_2_ enrichment could exacerbate lipid peroxidation in *M. lupulina*, but not in *L. perenne* plants, with no rising-CO_2_ responses in the ascorbate peroxidase (APX) and peroxidase (POX) in *M. lupulina*. No significant responses in the antioxidant enzyme activity, including APX, glutathione reductase (GR), POX, CAT, and SOD, were found when *B. vulgaris* plants were exposed to high levels of O_3_ with elevated CO_2_, except in the inhibition of APX (Kumari et al., 2013).

Instead, in *Quercus pubescens* and *Q. ilex* plants grown under elevated CO_2_, down-regulation of the protective systems was observed (Schwanz and Polle, 1998). According to the findings of Schwanz and Polle (1998), although the GR in oak leaves remains stable, the activities of SOD, CAT, POX, and APX, as well as the sum of dehydroascorbate and ASC, were reduced in CO_2_-elevated environments. Base on a recent report (Singh and Agrawal, 2015), the activities of the SOD, CAT, and APX declined, but the GR and POX were stimulated, finally leading to a significant reduction in the $O_{2}^{\cdot -}$, H_2_O_2_, and malondialdehyde (MDA) contents in *C. roseus* plants grown under elevated CO_2_. Recently, marked decreases in the ROS levels ($O_{2}^{\cdot -}$, H_2_O_2_) and reductions in some antioxidant enzymes, such as CAT and SOD, were observed simultaneously in mung bean plants exposed to elevated CO_2_, suggesting that a lower level of ROS might match the lower activity of antioxidant enzymes (Mishra and Agrawal, 2014).

Based on a report by AbdElgawad et al. (2015), who used C_3_ grasses (*L. perenne, Poa pratensis*) and C_3_ legumes (*M. lupulina*, *Lotus corniculatus*) as experimental materials, elevated CO_2_ can reduce the H_2_O_2_ level, lipid peroxidation, and lipoxygenase (LOX) activities, while it decreased the SOD, CAT, glutathione peroxidase (GPX), and GR levels, but did not affect the ASC-GSH cycle (AbdElgawad et al., 2015). Thus, the predominant form of the enzymatic antioxidant defense may strongly depend on the species and the abiotic stress (Duarte et al., 2013; Singh and Agrawal, 2015).

The activities and gene transcription expression levels of ROS scavenging enzymes in *A. thaliana* at elevated CO_2_ remained unchanged, particularly under well-watered conditions (Zinta et al., 2014). However, the excessive gene transcriptional response related to antioxidant metabolism due to O_3_ pollution was partly repressed by elevated CO_2_ in soybean (*Glycine max*) plants under field conditions, again arguing the protective role of elevated CO_2_ (Gillespie et al., 2012). Lipid peroxidation, indicated by MDA accumulation, would be lessened by CO_2_ enrichment, especially under other severe abiotic stress conditions such as drought (Salazar-Parra et al., 2012; Xu et al., 2014; AbdElgawad et al., 2015), heat wave stress (Xu et al., 2014; Zinta et al., 2014; AbdElgawad et al., 2015), O_3_ pollution (Yan et al., 2010; Kumari et al., 2013), and salinity (Pérez-López et al., 2009), implying that oxidative stress induced by severe environmental constraints may be mitigated, generally or at least partly, by CO_2_ fertilization. It is again highlighted that the positive vs. negative roles of elevated CO_2_ concentrations in antioxidant enzyme regulation under severe stressful abiotic environments may depend considerably on different species (Schwanz and Polle, 1998, 2001; Guo et al., 2006; Kumari et al., 2013; Xu et al., 2014; Zinta et al., 2014).

Moreover, based on a recent report using wheat plants, with increasing sugar levels via CO_2_ enrichment, sugar-derived reactive carbonyls (RCs; aggressive by-products of oxidative stress), including methylglyoxal (MG), were provoked by elevated CO_2_, which can negate the functions of multiple proteins, and impair the biological membrane, suggesting that plant diabetes may be inducible (Takagi et al., 2014), supporting an earlier study by Schwanz and Polle (1998). Thus, whether and how much elevated CO_2_ affects antioxidant systems in plant tissues depends on the plant species, crop variety, developmental stage, abiotic factors, and the combination of these (e.g., Hodges and Forney, 2000; Gill and Tuteja, 2010; Mishra and Agrawal, 2014; Xu et al., 2014; Zinta et al., 2014). This is a debatable issue, requiring further research.

### Response of Crucial Metabolites to Elevated CO_2_

The metabolism changes of certain important metabolites, and the related genetic variations induced by elevated CO_2_ have been found in a number of research reports. For instance, an accumulation of carbon compounds under elevated CO_2_ occurs in wheat leaves accompanied by an up-regulation of phosphoglycerate mutase (PGAM) involving carbohydrate transport, but a down-regulation of the adenosine diphosphate glucose pyrophosphatase protein for synthesizing starch; thus affecting the carbon flux within the plants’ tissues, and the balance between the carbon sink and source (Aranjuelo et al., 2011). Changes in the major chemical components induced by elevated CO_2_ have also been reported in many studies. Generally, under elevated CO_2_, there may be a decrease in the total N and organic N compounds, which define the elevated CO_2_-induced dilution effectiveness. However, there is an increase in the total non-structural carbohydrates (TNC), including starch and sugar (e.g., glucose, fructose, sucrose; Lavola and Julkunen-Tiitto, 1994; Poorter et al., 1997; Luo et al., 2004; Markelz et al., 2014), with a mostly stable level in the total structural carbohydrates (cellulose plus hemicellulose), lignin, and lipids (review by Poorter et al., 1997; Markelz et al., 2014). Nitrogen assimilation may be enhanced by elevated CO_2_ (Ribeiro et al., 2012), and a recent report indicated that elevated CO_2_ may promote N assimilation and transamination-related enzyme activities, such as glutamate oxoglutarate aminotransferase (GOGAT) and glutamate oxalate transaminase (GOT), and lead to an increase in the phloem amino acid content in *M. truncatula* (Guo et al., 2013).

Elevated CO_2_ can change not only the primary metabolic processes, but also the secondary metabolic composition in plant tissues (Lavola and Julkunen-Tiitto, 1994; Poorter et al., 1997; Matros et al., 2006; Lavola et al., 2013). Here, we mainly address the secondary metabolite responses, because there are fewer studies related to the key metabolites. Plant secondary metabolites often indicate that these compounds have no primary functions in the maintenance of life processes in plants; however, they are involved in the biological processes of plants dealing with environmental stress, with regard to adaptation and defense (Lavola and Julkunen-Tiitto, 1994; Ramakrishna and Ravishankar, 2011). Changes in the secondary metabolites with rising CO_2_ have been reported in several relevant studies. For example, an alteration in the carbon allocation under elevated CO_2_ has revised the carbon-nutrient balance (CNB) hypothesis (Bryant et al., 1983), increasing the C:N ratio in plant tissues (e.g., Poorter et al., 1997; Cotrufo et al., 1998; Xu et al., 2007), while increasing the levels of the C-based secondary compounds due to easier synthesis, in plants with excess carbon relative to the other nutrients (such as N; Lavola and Julkunen-Tiitto, 1994; Matros et al., 2006). However, a contradiction may arise when elevated CO_2_-induced N dilution limits the carbohydrate reserves, leading instead to a reduction in some secondary substances (Lavola and Julkunen-Tiitto, 1994). However, with rising CO_2_, a large accumulation of some secondary metabolites, including phenylpropanoids, tannins, triterpenoids, phenolic acids, and alkaloids, was observed, despite the effectiveness of the dilution (Lavola and Julkunen-Tiitto, 1994; Matros et al., 2006; Ghasemzadeh et al., 2010; Lavola et al., 2013). For example, in tobacco leaves there was a large accumulation of phenylpropanoids, including the major carbon-rich compound chlorogenic acid (CGA), and the scopolin and scopoletin coumarins (Matros et al., 2006). In the flavonoid response, although both the kaempferol and fisetin were increased by elevated CO_2_ in ginger (*Zingiber officinale* Roscoe; Ghasemzadeh et al., 2010), whether there was a decrease or increase in birch plants grown in elevated CO_2_ depended on the genetic type or environmental conditions (Lavola and Julkunen-Tiitto, 1994; Lavola et al., 2013). The glucosinolate accumulation was enhanced in *Brassica* plants exposed to elevated CO_2_, possibly changing the feeding behavior of specialized herbivores (Klaiber et al., 2013). In other metabolites, including lignin, cell wall polysaccharides, and terpenes, no obvious response was found, depending on the compound composition, species, genotype, nutrient status (such as N availability), and other environmental factors (Poorter et al., 1997; Lindroth et al., 2001; Matros et al., 2006; Lavola et al., 2013; AbdElgawad et al., 2014; Singh and Agrawal, 2015). For example, the levels of the condensed tannins, most flavonols, and phenolic acids in birch plants can be stimulated by elevated CO_2_ and elevated UVB, but this effect disappeared at high temperatures (Lavola et al., 2013).

**FIGURE 3:**
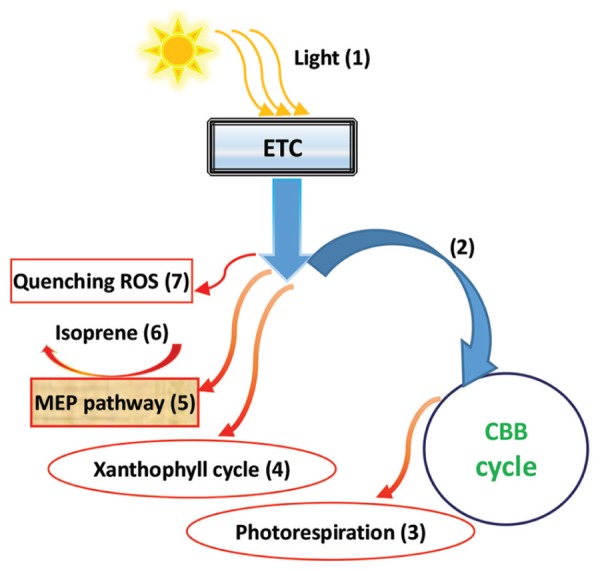
**A diagrammatic representation of isoprene biosynthesis downregulation in response to elevated CO_2_**. Light energy from the sun (1) is transferred into the plant metabolic bioprocesses using an electron transport chain (ETC). Under elevated CO_2_, more electron flux may be used for the CBB cycle for photosynthesis (2), while less electrons may flow into the photorespiration cycle (3), xanthophyll cycle (4), and the methylerythritol 4-phosphate pathway (MEP), (5) to synthesize isoprene (6), as well as other redox reactions, including the quenchers of ROS (7) (based mainly on Morfopoulos et al., 2014).

Isoprene is a volatile hydrocarbon molecule, generally emitted by certain vegetation types, particularly tree species, protecting plants against damage from abiotic stress, and playing an important role in tropospheric chemistry and climate change due to its highly reactive molecular properties, especially in the formation processes of ozone and secondary organic aerosols (Sharkey and Singsaas, 1995; Claeys et al., 2004; Sun et al., 2012). However, it has been confirmed that isoprene may have an active function in protecting the photosynthetic apparatus against oxidative stress from abiotic stress (such as heat), by quenching the ROS via the promotion of oxidative defense machinery (Gill and Tuteja, 2010; Morfopoulos et al., 2014). In a number of related reports, elevated CO_2_ has produced various effects on plant-derived isoprene emissions, including increases (Sharkey et al., 1991; Tognetti et al., 1998), remaining unchanged (Rosenstiel et al., 2003; Sun et al., 2012), and, most often, showing decreases (e.g., Wilkinson et al., 2009; Possell and Hewitt, 2011; Morfopoulos et al., 2014). The reason for the decreasing isoprene emission may be that the available reducing power captured by light may cause a large consumption, due to carbon fixation rather than isoprene synthesis, in CO_2_ enrichment conditions, resulting in a reduction in isoprene emissions (Morfopoulos et al., 2014). A reduction in the isoprene emission capacity may be attributable to a decrease in both the isoprene synthase activity and pool size of dimethylallyldiphosphate (DMADP), an immediate isoprene precursor (Sun et al., 2012). Actually, DMADP synthesis is involved in the primary photosynthetic product of glyceraldehyde-3-phosphate (GAP), linked to a leaf’s photosynthetic carbon metabolism (Loreto and Sharkey, 1993; Lichtenthaler, 1999; Sun et al., 2012; Trowbridge et al., 2012). Reduced ATP induced by elevated CO_2_ may also diminish DMADP synthesis (Sun et al., 2012). Thus, the isoprene emission capacity may be determined by the status of the balance between the primary metabolites, such as sugar, and the secondary metabolites, such as isoprene (Loreto and Sharkey, 1993; Sun et al., 2012), again highlighting the importance of the primary-secondary metabolite balance (abbreviated by PSMB) with CO_2_ enrichment. Based on the response model suggested by Morfopoulos et al. (2014), **Figure 3** succinctly describes a pathway involved in the downregulation of isoprene biosynthesis in response to elevated CO_2_. Under elevated CO_2_, more electron flux may be used in the CBB cycle for photosynthesis, whereas less electrons may flow into the photorespiration cycle, xanthophyll cycle, and the methylerythritol 4-phosphate (MEP, 5) pathway to synthesize isoprene, as well as other redox reactions, such as quenching ROS (e.g., GR reaction demands of NADPH; Gill and Tuteja, 2010). It is worth noting that the isoprene biosynthesis and emission, in and from plants, may be tightly associated with photosynthesis, photorespiration, the xanthophyll cycle, and oxidative defense systems in response to CO_2_ enrichment, with abiotic environmental changes (Gill and Tuteja, 2010; Moroney et al., 2013; Morfopoulos et al., 2014; **Figure 3**).

The role of hormone pathways in regulating the growth and metabolic responses to elevated CO_2_ is not well known, despite there being a few reports (e.g., Li et al., 2011; Ribeiro et al., 2012; Zavala et al., 2013). Elevated CO_2_ can promote an accumulation in the salicylic acid (SA, Zavala et al., 2013) and brassinosteroids (BR; Jiang et al., 2012), while reducing Jasmonates (JA) and ethylene concentrations (Zavala et al., 2013; Vaughan et al., 2014). Elevated CO_2_-induced genes were associated with the metabolic processes of the BR regulator in plant tissues (Li et al., 2006), which can alleviate the heat-induced inhibition of photosynthesis, by increasing the carboxylation efficiency and enhancing the antioxidant systems in *Lycopersicon esculentum* (Ogweno et al., 2008). In one recent report, BRs were found to enhance the stimulation of plant growth and photosynthetic potential under elevated CO_2_ (Jiang et al., 2012). An increase in the indole-3-acetic acid (IAA), isopentenyl-adenosine (iPA), and dihydrozeatin riboside (DHZR) was found, while a decrease in the ABA and unchangeable zeatin riboside (ZR) occurred in *Pinus tabulaeformis* plants exposed to elevated CO_2_, which can encounter O_3_ exposure effects to alleviate damage (Li et al., 2011). The iPA, DHZR, and ZR are recognized as the most commonly active cytokinins (CTKs) in plants. The results of the experiment by Ribeiro et al. (2012) indicated that elevated CO_2_ may play a role similar to gibberellin (GA) in the integration of carbohydrate and nitrogen metabolisms underlying the optimal biomass determination. When the *Arabidopsis* plants exhibited the inhibition of growth via the GA biosynthesis inhibitor (low-GA regime), the activities of the enzymes involved in photosynthesis, including the CBB cycle enzymes [phosphoglycerate kinase (PGK) and transketolase (TK)], were enhanced by elevated CO_2_, whereas the activities of the enzymes related to organic acid metabolism, such as the NAD-dependent malate dehydrogenase (MDH), were inhibited (Ribeiro et al., 2012). Moreover, nitrate reductase (NR) can be stimulated by elevated CO_2_ (by 31%) in plants with a low-GA content, indicating that rising CO_2_ may mediate inorganic N metabolism in association with GA (Ribeiro et al., 2012). This clearly indicates that elevated CO_2_ can substitute for the relevant metabolic bioprocesses in the low-GA species, which may have a marked potential application for plants, particularly staple crops, to cope with future climate change in a high CO_2_ concentration environment.

## General Gene Expression Profile Under Elevated CO_2_

The genes expressed differently between ambient and elevated CO_2_ might encode great changes in their metabolic functions (Li et al., 2006), including increases in the expression of a subset of genes encoding stress-related functions, and decreases in the expression of genes encoding chloroplast functions and other processes of photosynthesis (Moore et al., 1999; Miyazaki et al., 2004; Li et al., 2006). The decline in the gene expression may partly lead to so-called photosynthetic acclimation to long-term elevated CO_2_, particularly under limited environmental conditions or in carbon sink limited species (Jifon and Wolfe, 2002; Ainsworth et al., 2004; Long et al., 2004; Fukayama et al., 2012; Xu et al., 2013a). For example, Fukayama et al. (2012) found the overexpression of Rubisco activase in rice leaves grown under elevated CO_2_, possibly leading to a decrease in the photosynthetic capacity. However, the gene expression in response to CO_2_ fumigation may depend on different developmental stages at the time of sampling, and different physiological conditions of the ecotypes of *A. thaliana* (Li et al., 2006). Moreover, limited N, a typical example of a nutrition resource deficit, may lower the stimulation of photosynthesis by elevated CO_2_, due to excess photoassimilate availability, triggering sugar-signaling feedback. This reduces the expression of the photosynthetic genes, especially in Rubisco, leading to the allocation of photosynthetic N into sinks that are more necessary for relative biosynthesis (Moore et al., 1999; Leakey et al., 2009b; Markelz et al., 2014). Based on a report by Duanmu et al. (2009), the enhanced expression of the limited CO_2_-induced gene *HLA3* may increase the HCO_3_^-^ transport and photosynthetic *C*_i_ affinity, which may counter the down-regulation of the photosynthetic capacity under CO_2_ enrichment, if the gene can be transported to higher plants (Price et al., 2011). This demonstrates the potential modified gene applications in the improvement of photosynthetic regulation traits in high CO_2_ climates.

In a rice cultivar, gene expression for D1 protein (a protein of PSII gene) was down-regulated by 20% at heat stress under elevated CO_2_, but this change did not occur in another cultivar, indicated that elevated CO_2_ may enhance the damage of D1 protein, depending on genotypic variation (Gesch et al., 2003). Based on a recent study in poplar plants (Liu et al., 2014), only eight significantly changed key genes involved in crucial metabolisms in response to elevated CO_2_ were identified by a qRT-PCR test. During wheat plant senescence, up-regulation of genes related to nitrogen remobilization, and down-regulation of genes related to carbon remobilization were observed under elevated CO_2_, reflecting greater grain N-sink strength of developing grains (Buchner et al., 2015). Based on a microarray analysis, the *A. thaliana* photosynthetic gene expression can be most adversely affected by abiotic stress, such as heat and drought, where almost all of genes were down-regulated. However, the greatest down-regulation in gene expression can be diminished by elevated CO_2_ (Zinta et al., 2014). From the genome-wide expression profiling of the mRNA in *A. thaliana* leaves (Zinta et al., 2014), 3643 differentially expressed genes appeared between plants exposed to climate extremes and ambient CO_2_, whereas only 2841 genes were obtained when grown under elevated CO_2_. Specifically, both the up-regulated and down-regulated genes were remarkably lower in plants exposed to elevated CO_2_, than in ambient CO_2_. For example, under stressful conditions such as heat and drought, the down-regulations of the genes involved in the light reactions (photosystem I and II, light-harvesting complex II), pigment synthesis, and the Calvin cycle can be dampened by elevated CO_2_, being consistent with changes in photosynthetic rates. It is indicated that elevated CO_2_ may repress the impact of climate extremes on gene expression in rosette leaves (Zinta et al., 2014). It is worth noting that we only presented a general description here, and a detailed list of the gene expression differences may be found in the report by Zinta et al. (2014).

Elevated CO_2_ often can induce a marked decline in photorespiration (see above), suggesting that there may be an involvement of the expression of the genes related to photorespiration pathway including both transcripts and metabolite levels (Sharkey, 1988; Novitskaya et al., 2002; Foyer et al., 2009; Florian et al., 2014; Wang et al., 2014). A *BOUT DE SOUFFLE* (*BOU*) gene encoding a mitochondrial carrier may be involved in photorespiration in *Arabidopsis* because of the knockout mutant *bou-2* can arrest growth at ambient CO_2_, but not at high CO_2_ concentration, implying *BOU* gene linking glycine decarboxylase (GDC) activity, may regulate the response to CO_2_ concentration changes (Eisenhut et al., 2013). Plants defective (*glyk1* mutants), the gene encoding glycerate kinase (GLYK), cannot grow in normal CO_2_ level but fully recover at elevated CO_2_, which the reasonable reason why the mutant requires a high CO_2_ concentration is unknown (Timm and Bauwe, 2013). The transcript levels of some photorespiratory genes up-regulated such as plastid chaperonin proteins (CPN60B), and those down-regulated such as GDC under heat and drought stresses, were largely repressed under elevated CO_2_, but that is not universal for all genes (Zinta et al., 2014). Furthermore, according to a study by Florian et al. (2014), the transcript levels of photorespiratory genes in *Arabidopsis* were almost unchanged at high CO_2_ concentration except a decline in transcript levels of glycine decarboxylase H-protein (GDCH1) that functions in photorespiratory carbon recovery. Thus, whether and how the photorespiratory gene expression play a major role in responses to atmospheric CO_2_ concentration changes are mostly unknown (Foyer et al., 2009; Timm and Bauwe, 2013; Florian et al., 2014), which needs to be tested further.

Because antioxidant defense systems would be enhanced by elevated CO_2_, the gene expression levels of antioxidant enzymes may be also promoted accordingly (Gillespie et al., 2012; Mishra and Agrawal, 2014; Zinta et al., 2014). In *A. thaliana* plants, CO_2_ enrichment can up-regulate the gene transcriptional expression of an antioxidant enzyme, dehydroascorbate reductase (DHAR), but down-regulate that of CAT, particularly under stressful environments. However, the gene expression changes in others such as APX, GR, GPX, POX, and SOD to elevated CO_2_ were not significant (Zinta et al., 2014). Additionally, a high transcript abundance for the majority of the genes coding antioxidant recycling enzymes enhanced by high O_3_ concentration was also not affected by elevated CO_2_ (Gillespie et al., 2012). Elevated CO_2_ did not modify the up-regulation of transcripts of oxidative-stress-related genes induced by herbivory or elevated O_3_ in soybean plants (Casteel et al., 2008). Kontunen-Soppela et al. (2010) indicating that CO_2_ enrichment cannot alleviate harmful effects from O_3_ pollution based on a gene expression test in birch plants. Thus, the authors could not conclude that CO_2_ enrichment can up-regulate the gene transcriptional expression levels of the antioxidant enzymes under stressful environment. Further studies are needed urgently to elucidate the molecular responses in the diverse antioxidant systems in responses to elevated CO_2_ with the key environmental factors including drought, heat, and ozone (Gillespie et al., 2012; Zinta et al., 2014).

One recent research study described the results of a gene bioinformatics analysis of hardy winter wheat (*Triticum aestivum*), with different low temperature adaptive capacities in response to elevated CO_2_ (Kane et al., 2013). The genes induced by elevated CO_2_ was three times higher in the non-acclimated (NA) relative to cold-acclimated (CA) conditions (1,022 vs. 372). The greatest down-regulation of genes appeared in the plant defense responses in the NA plants. On the other hand, CA can reverse this down-regulation, due to the cold-induced genes involved in the plant’s resistance to pathogenesis, and cellular and chloroplast protection (Kane et al., 2013), suggesting that cold-adapted hardy winter plants may be less affected by elevated CO_2_. Conversely, the plants that are more sensitive to cold weather may be regulated both easily and drastically via CO_2_ enrichment. Of note is the down-regulative interaction of high CO_2_ levels with low temperature adaptations, which requires further investigation.

Another recent microarray study describes the expression of the respiratory genes in *A. thaliana* plants exposed to elevated CO_2_, with both limited and sufficient N availabilities (Markelz et al., 2014). This analysis showed that 4439 transcripts were significantly different between the ambient and elevated CO_2_. Particularly, the transcriptional response of the genes related to protein synthesis was greatest during the day, due to elevated CO_2_ induction. These genes included those related to the components of glycolysis, the TCA cycle, the mitochondrial electron transport chain (ETC), and the mitochondrial protein import complexes. The evidence of the up-regulation of the transcription of the genes, with relation to respiration under elevated CO_2_ levels, has also been obtained from rice (Fukayama et al., 2011) and soybean plants (Leakey et al., 2009b). Furthermore, 1,708 transcripts differed significantly in abundance between the limited N and ample N availabilities, while 258 transcripts differed significantly due to the interactions of the CO_2_ level and N availability, again indicating that the expression of the genes related to the key physiological bioprocesses in response to elevated CO_2_ may be markedly affected by other environmental factors, such as N limitation (Markelz et al., 2014) and day length (Queval et al., 2012). It is worth pointing out that the systematicness and complexity of the underlying molecular mechanisms may coexist in the plant response to elevated CO_2_, and its interaction with other multiple abiotic factors including nutrition condition.

In addition to the relevant studies concerning the specific gene manipulation and genome-wide transcriptional analysis, with strong selective pressure due to the novel CO_2_ level, the evolutionary adaption to an atmospheric CO_2_ concentration change has been found in many reports of the stomatal developmental response (Gray et al., 2000; Ward and Kelly, 2004). Moreover, because the previous studies concerning the response to CO_2_ enrichment are often limited to one generation of the plant life-cycle (Ward and Kelly, 2004), to further understand the genetic variations in the plants exposed to long-term elevated CO_2_, Teng et al. (2009) found that the maternal genetic effects of elevated CO_2_ cannot be retrieved in their offspring after undergoing 15 generations of *A. thaliana* grown in a long-term elevated CO_2_ atmosphere, indicating the lack of genetic variation and specific adaptations for CO_2_-enriched responsiveness (Teng et al., 2009). It is suggested that selective pressure from elevated CO_2_ may be not enough to produce a genetic modification to adapt to new environmental changes. This issue should be investigated further, with long-term exposure to elevated CO_2_.

## Elevated CO_2_ Interactions with Multiple Abiotic Stresses

There have been several review reports concerning the interactions between elevated CO_2_ and other abiotic factors, such as temperature or drought, on plant growth and physiological processes (e.g., Morison and Lawlor, 1999; Ainsworth and Rogers, 2007; Peñuelas et al., 2013; Ruiz-Vera et al., 2013). However, the underlying mechanism concerning the responses to CO_2_ enrichment with multiple factors has rarely been systematically reviewed (Xu et al., 2013a; Jagadish et al., 2014; Way et al., 2015). Although the related descriptions have been presented in the appropriate places above, here, we present a succinct statement, particularly for the underlying mechanisms in physiological responses to elevated CO_2_, in combination with several abiotic factors, such as drought and heat waves.

Water deficits and heat waves are considered to be the most critical stress factors with markedly potential effects on plant growth, crop yield, vegetation productivity, photosynthetic capacity, promotion of ROS accumulation (such as H_2_O_2_), and the oxidative enhancement of functional molecules, such as active proteins and DNA (e.g., Mittler, 2006; Xu and Zhou, 2006; Barnabás et al., 2008; Xu et al., 2009b, 2013a, 2014; Zinta et al., 2014). With the exception of a few reports (e.g., Coleman et al., 1991; Roden and Ball, 1996), most of the relevant studies have concluded that CO_2_ fertilization may mitigate the adverse impacts of environmental stresses, such as heat, drought, O_3_ pollution, and their combinations (Biswas et al., 2013; Xu et al., 2013a, 2014; Zinta et al., 2014). These aspects of the mitigation of CO_2_ enrichment include relatively increased individual growth (e.g., Xu et al., 2014) and community production (Naudts et al., 2014), enhanced photosynthesis (Biswas et al., 2013; Xu et al., 2014; Zinta et al., 2014), elevated WUE and NUE (Palmroth et al., 2013), optimized chlorophyll fluorescence (Biswas et al., 2013; Xu et al., 2014; Zinta et al., 2014), up-regulated antioxidant defense metabolism via increased lipophilic antioxidants and membrane-protecting enzymes (Naudts et al., 2014; Xu et al., 2014), and decreased photorespiration with low H_2_O_2_ production (Foyer and Noctor, 2009; Munne-Bosch et al., 2013; Zinta et al., 2014).

Elevated CO_2_ may help the leaf tissues of a dominant grass in Northern China to partly escape the negative effects of heat and drought stresses on plant growth, canopy structure, leaf development, photosynthetic potential, and antioxidant systems (Xu et al., 2014). For *A. thaliana* plants, the combination of the heat and drought-induced inhibition of photosynthesis was 62% under ambient CO_2_, but the reduction in photosynthesis was only 40% with elevated CO_2_. Furthermore, the protein carbonyl content, a marker of protein oxidation, increased significantly during a heat wave and drought, in which the effects were repressed by increased CO_2_ (Zinta et al., 2014). The dramatic differences between the altered transcriptional expression of *A. thaliana* plants subjected to a combination of heat and drought stresses were demonstrated in the presence and absence of elevated CO_2_, with less down-regulation of the genes involved in the light reactions (photosystem I and II, light-harvesting complex II), pigment synthesis, and the CBB under elevated CO_2_. Additionally, there was less limitation to the photosynthetic parameters, such as *A*_net_, maximum photochemical efficiency (*F*_v_/*F*_m_), *g*_s_, and chlorophyll content (Zinta et al., 2014), possibly due to the effectiveness of the mitigation of the CO_2_ enrichment. Moreover, following cancelation of the extreme heat and drought stresses, the plant growth and physiological activities related to the positive responses to growth may partly resume at high CO_2_ concentrations, and the oxidative stress can be greatly alleviated, although they cannot reach the control levels (Xu et al., 2009a, 2010; Xu and Zhou, 2011; Zinta et al., 2014). Again, this implies that CO_2_ fertilization may alleviate the damage of extreme climatic events, such as snap heat waves and droughts, compromising part of the loss and accelerating recovery in case of the elimination of severe abiotic stress. However, a recent report indicated that high temperature, with no elevated CO_2_, provokes the drought sensitivity of the leaf to gas exchange, while the latter did not affect the *Eucalyptus radiata* seedling response to drought, and cannot alleviate the negative effects of rising temperature on drought stress (Duan et al., 2014). From another point of view, drought, warming, air pollution, and, particularly, their combination may substantially negate the elevated-CO_2_ stimulation in photosynthesis, plant growth, and productivity (Biswas et al., 2013; Ruiz-Vera et al., 2013; Xu et al., 2013a; Duan et al., 2014), which is worth noting.

A hot issue has arisen, in which the photosynthetic responses to elevated CO_2_ and its combination with climatic change may differ completely between plant species within their photosynthetic pathways. Because of the C_4_ specific photosynthetic pathway with a CO_2_-concentrating pump (von Caemmerer and Furbank, 2003), they cannot benefit from elevated CO_2_ relative to C_3_ plants (Leakey et al., 2006; Morgan et al., 2011; Bütof et al., 2012; Xu et al., 2014). However, there is a practical and explicable positive response of growth and photosynthesis to elevated CO_2_, with drought and heat stress in C_4_ plants. (1) Although no obvious response to CO_2_ enrichment occurs under ample water availability, great stimulation in the growth and photosynthetic capacity may be obtained under water deficits, due to the marked alleviations of drought stress via *g*_s_ reduction and WUE elevation, and oxidative stress mitigation under elevated CO_2_ (e.g., Long et al., 2004; Ghasemzadeh et al., 2010). (2) The higher temperature might benefit the C_4_ species that originate from, and currently grow under warming conditions, and because the photorespiration of C_3_ plants increases with rising temperature, leading to a reduction in the *A*_net_. The C_4_ plants lack photorespiration pathways, with no effect on photosynthesis (Long et al., 2006; Long and Ort, 2010; Morgan et al., 2011). Actually, both hypotheses have been well tested in several reports (Morgan et al., 2001, 2011; Leakey et al., 2006; Xu et al., 2014). This positive response to a combination of CO_2_ enrichment and warming, as well as water deficits, highlights the fact that C_4_ plants may have a great potential advantage in future climatic change. A higher CO_2_ concentration with warming and drought suggests that C_4_ plants may prosper in these vulnerable ecosystems in arid and semiarid regions in the future (Morgan et al., 2011; Lobell et al., 2013; Xu et al., 2014). However, elevated CO_2_ induced an electron transfer rate (ETR) enhancement in one C_3_ species, *Halimione portulacoides*, and one C_4_ species, *Spartina maritime*, but with lower photosynthetic efficiency in the C_4_ plants due to an increase in the dissipated energy flux, indicated by higher non-photochemical quenching (NPQ), suggesting that the abundance of C_3_ species may increase in Mediterranean halophyte vegetation (Duarte et al., 2014). Thus, the future climatic change may induce a rapid shift in some terrestrial vegetation, because of the different responses between the species with the specific photosynthetic pathways, such as C_3_ and C_4_ plants, depending on the combination of multiple climatic factors.

## Conclusion

We briefly summarize several key points. (1) Elevated CO_2_ generally increases the *A*_net_, in which the positive responses strongly depend on the plant functional groups and species, with the expected stimulation from rising CO_2_, for almost all of the C_3_ species, but only for C_4_ plants under water deficit conditions (due to the CCM). The performance of Rubisco in fixing carbon is promoted by CO_2_ enrichment, because of its dual character. However, a downregulation in the photosynthetic capacity may occur because of the decreased ATP:ADP ratio, diluted N, and excessive photosynthate accumulation under continually rising CO_2_, particularly under N and/or carbon sink limitations. (2) An elevated CO_2_-induced suppression of photorespiration has been tested using a lower Gly:Ser ratio as an indicator, while a general negative response in mitochondrial respiration varies, depending on the species. The balance between the increased respiratory substrate and diluted N may play a key role in the rising CO_2_-induced response, with evidence from the expression up-regulation of the genes related to the respiratory pathway. (3) Plants may run an antioxidant defense system with both the enzymatic and non-enzymatic machinery protected from the damage of oxidative stress due to the generation of ROS under abiotic stresses (such as drought and heat), while elevated CO_2_ may partly promote the accumulation of antioxidants like polyphenols and ascorbate, and enhance some antioxidant enzyme activities to diminish the oxidative stress from abiotic factors, alone or combination, depending on the genetic variations and plant developmental stage. (4) Elevated CO_2_ leads to a lower N level and higher content of the total non-structural carbohydrates (TNC), including starch and sugars, while remaining mostly stable in the totals of the structural carbohydrates, lignin, and lipids. However, some secondary metabolites, such as phenylpropanoids, tannins, and phenolic acids, are enhanced by CO_2_ enrichment. Isoprene emissions may be weakened by elevated CO_2_, because biosynthesis may need to balance the ATP and NADPH with photosynthetic metabolism. (5) Elevated CO_2_ might mitigate the adverse effects of abiotic stresses via relatively increased individual growth, enhanced photosynthesis, increased resource use efficiency, promoted antioxidant defense metabolism, and decreased photorespiration under multiple environmental stresses. In terms of the photosynthetic pathway, CO_2_ enrichment did not affect C_4_ plants under ample environmental conditions, but promoted it when exposed to drought, warming and their combination, predicting a great potential advantage in future climatic change scenarios for the C_4_ species, particularly in arid and semiarid areas.

## Future Perspectives

### Promotion of the Relevant Research

In the future, we may focus on several crucial research aspects: (1) to further elucidate the underlying mechanisms of the response to CO_2_ enrichment in key biological processes, including photosynthesis, antioxidant machinery, and other related critical metabolic bioprocesses, such as hormone-involved regulation, as well as the relevant biochemical signal cascades; (2) to disentangle and compare the diverse responses from different species and PFTs to elevated CO_2_ or its combination with other abiotic factors; (3) to integrate various spatial-temporal scales from molecular, cellular, biochemical, physiological, individual, ecosystem, and global vegetation levels, and from instantaneous to annual or longer time-scales to elucidate the underlying genetic mechanisms in association with key biological processes under the effects of global environmental factors, including elevated CO_2_, warming, drought, and air pollution; (4) to strengthen the linkages to other relevant research subjects, including ecological, biogeoscience, environmental, climatic, and social-economic aspects, to find appropriate synthetic solutions to urgent practical issues like environmental contamination, ecosystem damage, and global warming impacts.

### Potential Applications under Future Climate Change

Future climate change may impact key biological metabolic processes and their feedback. For example, environmental stresses may provoke the generation of ROS in chloroplasts, the site of photosynthesis, while future high CO_2_ levels may alleviate the limitations of these stresses. We might also use biotechnological tools such as the protection function against ROS to deal with future climatic change. In addition, Rubisco properties may be improved by regulating the transgenic expression of Rubisco activase in crops such as rice, possibly enhancing the photosynthetic capacity under rising CO_2_ (Fukayama et al., 2012), while the high CO_2_-induced downregulation of the photosynthetic capacity might induce the modification of the photosynthetic pathway (Price et al., 2011). Furthermore, the modified genetic capacity for the high utilization of photosynthate to strengthen sink storage may make plants capable of sustaining increased photosynthesis when the plants are grown in elevated atmospheric CO_2_, while additional thermo-tolerant transgenic crops may be required to cope simultaneously with climatic warming (Lavania et al., 2015). Finally, research should be conducted to strengthen the feasible applications from the relevant research results in response to CO_2_ enrichment, and its combination with multiple environmental factors for ecological management, climate change mitigation, sustainable development, and related policy decisions, but not at the expense of environment.

## Author Contributions

YJ is co-first author, ZX and YJ collected and analyzed the data, ZX, YJ, and GZ wrote the manuscript.

## Conflict of Interest Statement

The authors declare that the research was conducted in the absence of any commercial or financial relationships that could be construed as a potential conflict of interest.
